# Dietary pattern analysis among stone formers: resemblance to a DASH-style diet

**DOI:** 10.1590/2175-8239-JBN-2019-0183

**Published:** 2020-06-01

**Authors:** Fernanda Guedes Rodrigues, Thalita Melo Lima, Lysien Zambrano, Ita Pfeferman Heilberg

**Affiliations:** 1 Universidade Federal de São Paulo, Programa de Nutrição, São Paulo, Brasil.; 2 Universidade Federal de São Paulo, Divisão de Nefrologia, São Paulo, Brasil.

**Keywords:** Nephrolithiasis, Diet, Nutritional Status, DASH Diet, Obesity, Nefrolitíase, Dieta, Estado Nutricional, Dieta DASH, Obesidade

## Abstract

Recent epidemiological studies have shown that dietary patterns may have a more persistent impact on the risk of stone formation than single nutrients of the diet. Dietary Approaches to Stop Hypertension (DASH), a low-sodium and fruits/vegetables-rich diet, has been associated with a lower risk of nephrolithiasis, due to altered urinary biochemistry. This observational study aimed to investigate whether the dietary pattern of stone formers (SF) resembled a DASH-diet and its influence on urinary lithogenic parameters. Anthropometric data, fasting serum sample, 24-h urine samples, and a 3-day food intake record under an unrestricted diet were obtained from 222 SF and compared with 136 non-SF subjects (controls). The DASH-diet food portions were determined from the food records whereas intakes of sodium chloride (NaCl) and protein (protein equivalent of nitrogen appearance, PNA) were estimated from 24-hr urinary sodium and urea. A dietary profile close to a DASH-diet was not observed in any of the groups. NaCl intake and PNA were significantly higher in SF versus non-SF (12.0 ± 5.2 v.s. 10.1 ± 3.4 g/day, *p* = 0.01 and 1.8 ± 0.1 v.s. 1.4 ± 0.1 g/kg/day, *p* = 0.03). SF exhibited a positive correlation of NaCl intake and PNA with urinary calcium, oxalate and uric acid, and of PNA with urinary sodium. SF consumed more vegetables and legumes, but less fruits and low-fat dairy items than non-SF. The present series presented a dietary profile characterized by low calcium and high salt and protein contents, not reflecting an ideal DASH-style diet pattern.

## Resumo

Estudos epidemiológicos recentes mostraram que os padrões alimentares podem ter um impacto mais persistente sobre o risco de formação de cálculos do que apenas os nutrientes da dieta. A dieta DASH (Dietary Approaches to Stop Hypertension), uma dieta pobre em sódio e rica em frutas/vegetais, tem sido associada a um menor risco de nefrolitíase, devido às alterações metabólicas urinárias. Este estudo observacional teve como objetivo investigar se o padrão alimentar de pacientes litiásicos (Lit) se assemelhava a uma dieta estilo DASH e sua influência nos parâmetros litogênicos urinários. Dados antropométricos, amostra de soro em jejum, amostras de urina de 24 horas e um registro de ingestão alimentar de 3 dias sob dieta irrestrita foram obtidos de 222 Lit e comparados com 136 indivíduos não-Lit (controles). As porções de alimentos do padrão de dieta DASH foram determinadas a partir dos registros alimentares, enquanto a ingestão de cloreto de sódio (NaCl) e proteína (Equivalente Protéico do Aparecimento de Nitrogênio Urinário, PNA) foram estimadas a partir da uréia e sódio urinários de 24 horas. Um perfil alimentar próximo a uma dieta estilo DASH não foi observado em nenhum dos grupos. A ingestão de NaCl e PNA foram significativamente maiores nos Lit versus não-Lit (12,0 ± 5,2 v.s. 10,1 ± 3,4 g/dia, *p* = 0,01 e 1,8 ± 0,1 v.s. 1,4 ± 0,1 g/kg/dia, *p* = 0,03). Os Lit exibiram uma correlação positiva entre a ingestão de NaCl e PNA com cálcio, oxalato e ácido urinários, e de PNA com sódio urinário. Os Lit consumiram mais vegetais e leguminosas, mas menos frutas e lácteos com teor reduzido de gordura do que os não-Lit. A presente série apresentou um padrão alimentar caracterizado por reduzido consumo de cálcio e elevado em sal e proteínas, não refletindo um padrão ideal de dieta DASH.

## Introduction

Nephrolithiasis represents a common disorder with a lifetime cumulative incidence of 5-10% and with a progressively increasing prevalence worldwide^1^. This systemic condition results from the interaction of metabolic factors, genetic inheritance, and environmental exposure, and is associated with hypertension, coronary artery disease, metabolic syndrome, and diabetes mellitus^2^. Obesity, overweight, and weight gain have also been associated with an increased risk of stone formation^3^^,^ 4. Metabolic syndrome and insulin resistance, the consequences of a larger body size, may contribute to the development of kidney stones by increasing the urinary excretion of calcium, uric acid, and oxalate and decreasing urinary pH^3^^,^ 5.

Dietary intervention is an integral component of prevention of kidney stones, since diets rich in animal protein, sodium chloride, and low calcium and fluid intake confer a higher risk for stone formation^6^^,^ 7. Some dietary patterns have already been associated to the reduction of risk for nephrolithiasis, such as Dietary Approaches to Stop Hypertension (DASH)^8^.

The DASH diet is rich in fruits, vegetables, whole grains, nuts, and legumes, moderate in low-fat dairy products, and low in animal protein, sodium, sugars, and saturated fats. The protective effects of such diet appear to be due to the increase in urinary magnesium, citrate, potassium, and pH^8^. It has been shown that fruit intake increases urinary citrate levels^9^^,^ 10. Moreover, urinary citrate may increase because of the intake of alkaline, non-citrus, and potassium-rich fruits, with a high content of malate and citrate as well^11^.

Given the potential influence of nutritional conditions and diet upon lithogenesis, the present study aimed to perform a dietary pattern analysis, investigating the adequacy to a DASH diet in 222 stone formers (SF) and compare it with a control group consisting of 136 individuals without history of urinary stones (non-SF). The influence of the dietary pattern upon lithogenic parameters was also evaluated.

## Methods

### Study population

A total of 240 patients referred to the Nephrolithiasis Outpatient Clinic of the Universidade Federal de São Paulo (UNIFESP) because of an established diagnosis of renal stone were sequentially enrolled in the present cross-sectional and prospective study. The diagnosis of nephrolithiasis had been made based on the presence of renal colic with hematuria and spontaneous elimination and/or surgical/endoscopic removal of the stones and/or radiographic evidence of stones. Exclusion criteria were age < 18 years old, chronic kidney disease (estimated glomerular filtration rate < 60 mL/min/1.73m^2^), pregnancy, renal tubular acidosis, hyperparathyroidism, recurrent urinary tract infection, inflammatory bowel disease, intestinal resection/bariatric surgery, or malignant diseases. At the initial evaluation, anthropometric data including body mass index (BMI) and waist circumference (WC) were obtained and the patients were instructed to fill up a 3-day food-intake record under their regular unrestricted diet and collect a fasting serum and timed 24-h urine sample for determination of urinary risk factors. The 24-hr urine sample should be preferentially collected after completing the last (third) day of food record. When not possible, an interval of 1 week between the food record completion and the collection of the urine sample was acceptable. All patients were treatment-naïve with respect to stone disease. In case they were receiving thiazides for hypertension, they were oriented to withdraw it during the 72 hours before the collection of the 24-hr urine sample. A control group of 150 adult subjects without history of urinary stones, consisting of staff members or people accompanying patients who agreed on completing the dietary records and following the same aforementioned protocol, was considered for comparison. The study was conducted from May 2016 through May 2018.

The Local Medical Ethics and Research Committee approved the study and an informed consent was obtained from all subjects.

### Nutritional assessment

All participants were subjected to measurement of WC and body weight and height for the calculation of BMI. Patients were classified according to BMI into: normal weight (< 25 kg/m^2^), overweight (25 - 29.9 kg/m^2^), and obese (≥ 30 kg/m^2^). Dietary intake was evaluated from the 3-day food records. The participants were instructed to write down their total daily food intake close to the date of the 24-hr urine collection, either at the last (third) day of filling up the food diary or at a maximum interval of one week. The food intake was reported through home-based measures, describing the amount of each food consumed without changing current eating habits. After receiving the records, a nutritionist evaluated and corrected the food diary during an interview. These data were used to calculate nutrients using the Software Dietpro 6.0, which contains the tables of the US Department of Agriculture as the nutrient database. The DASH-style food portions were determined from the food records as well and based on the DASH diet guide^12^. Food groups have been classified according to the DASH diet eating pattern as belonging to: total grains; whole grains; vegetables; fruits; low-fat or fat-free milk and milk products; meats, poultry and fish; nuts, seeds, and legumes; fats and oils; sweets/added sugar and sodium. Oxalate intake was calculated based on the table from Harvard website, using the foods available on this database^13^. The 24-hr urine sample was employed to estimate sodium and protein intakes. The protein intake was calculated using the protein equivalent of nitrogen appearance (PNA) formula: PNA=(urinary urea nitrogen + [0.031 × weight]) × 6.25, where urinary urea nitrogen is (urinary urea/ 2.14 × urinary volume).

### Biochemical parameters

Blood samples were tested for creatinine, urea, phosphorus, uric acid, potassium, fasting glucose, total cholesterol, LDL, HDL, and triglycerides. In the 24-hr urine samples, urinary volume, calcium, creatinine, urea, uric acid, citrate, oxalate, sodium, potassium, magnesium, phosphorus, and pH were determined. Creatinine was determined according to the modified Jaffe’s reaction, by an isotope dilution mass spectrometry (ID-MS) traceable method. Urinary calcium, phosphorus, and magnesium were determined by a colorimetric method; urea, uric acid, citrate, and oxalate by an enzymatic method, and sodium/potassium by ion-selective electrode. Serum glucose, uric acid, total serum cholesterol, and HDL were determined by an automated enzymatic method. LDL was calculated using the Friedwald Equation. All biochemical parameters were measured in a Beckman Clinical Chemistry Analyzer (AU480-America Inc., Pennsylvania, USA) and urine pH by pHmeter (Micronal São Paulo, Brazil). Idiopathic hypercalciuria was defined by serum calcium within normal limits and 24-hour urinary excretion of calcium ≥ 250 or 300 mg/24hr (for females and males, respectively). Hyperuricosuria was considered as urinary uric acid > 750 or 800 mg/24hr (for females and males, respectively), hypocitraturia as urinary citrate < 320 mg/24hr, and hyperoxaluria as urinary oxalate > 45 mg/24hr.

### Statistical analysis

Variable distributions were evaluated by Kolmogorov-Smirnov test. Categorical variables were presented as absolute and relative frequencies. Normal and skewed continuous variables were presented as mean and standard deviation or median and interquartile range (IQR), as appropriate. Generalized linear models were performed to determine the differences between the 2 groups allowing age adjustments. Spearman’s test was used for correlations. Statistical significance was defined as *p* < 0.05. All statistical analyses were conducted using Statistical Package for Social Sciences for Windows version 18.0 (SPSS Inc., Chicago, IL, USA).

## Results

From the 240 eligible patients, 10 declined to participate in the study, 4 had not provided a proper collection of 24-hour urine samples, and 4 had not adequately filled up the food record. Among the initially recruited controls, 10 did not show up to deliver the 24-hour urine samples, 3 did not collect the sample properly, and 1 had not adequately completed the food record. Demographic data, clinical and laboratorial parameters are shown in Table 1. The percentage of women was significantly lower (53.2 vs. 68.4%) and mean age significantly higher (41.7 ± 12.5 versus 36.0 ± 12.5 yrs old) in SF than in non-SF group, with diabetes and hypertension more prevalent in the former. SF presented significantly higher BMI, WC, and percentages of overweight and obesity. Mean serum plasma glucose and triglycerides were significantly higher and serum potassium was significantly lower in SF when compared to non-SF. Regarding the urinary parameters, SF presented significantly higher mean values of calcium, sodium, and oxalate excretion and significantly lower values of urinary citrate. The distribution of metabolic disturbances among SF, isolated or in association, was: hypocitraturia (31.7%), hypercalciuria (28.4%), hyperuricosuria (19.9%), and hyperoxaluria (6.5%).

**Table 1:** Demographic, clinical, and laboratorial parameters of stone formers (SF) and non-stone formers (non-SF)

	Non-SF n = 136	SF n = 222	*p*
Male/Female [n (%)]	43 (31.6)/93 (68.4)	104 (46.8)/118 (53.2)	0.004
Age	36.0 ± 12.5	41.7 ± 12.5	< 0.001
BMI (kg/m^2^)	25.8 ± 4.9	28.3 ± 5.8	< 0.001
Eutrophic [n (%)]	67 (49.3%)	69 (31.1%)	0.54
Overweight [n (%)]	42 (30.9%)	80 (36.0%)	< 0.001
Obese [n (%)]	27 (19.9%)	67 (30.2%)	0.03
WC (cm)	89.7 ±14.0	96.5 ± 14.2	0.012
Diabetes [n (%)]	5 (3.7%)	26 (11.7%)	0.006
Hypertension [n (%)]	10 (7.4%)	57 (25.7%)	< 0.001
*Serum*			
Fasting glucose (mg/dL)	81.7 ± 9.9	96.6 ± 24.7	< 0.001
Urea (mg/dL)	28.4 (23.0 - 34.5)	29.9 (24.0 - 34.7)	0.22
Creatinine (mg/dL)	0.8 (0.6 - 0.9)	0.9 (0.7 - 1.0)	0.07
Uric acid (mg/dL)	4.9 (4.0 - 5.8)	5.2 (4.0 - 6.3)	0.38
Potassium (mg/dL)	4.5 (4.2 - 4.7)	4.3 (4.1 - 4.5)	0.02
Phosphorus (mg/dL)	3.4 (2.9 - 3.7)	3.3 (2.9 - 3.7)	0.50
Total cholesterol (mg/dL)	183 (156 - 209)	198 (166 - 224)	0.16
LDL cholesterol (mg/dL)	105 (80 - 126)	114 (91 - 138)	0.07
HDL cholesterol (mg/dL)	53.2 ± 12.8	52.2 ± 23.0	0.69
Triglycerides (mg/dL)	116 ± 88.0	159 ± 93.1	0.01
*Urine*			
Volume (mL)	1478 ± 691	1937 ± 763	< 0.001
Calcium (mg/24h)	158 ± 88	216 ± 118	0.01
Sodium (mEq/24h)	172 ± 62	206 ± 89	0.04
Potassium (mEq/24h)	49.0 (33.7 - 60.7)	50.0 (39.5 - 63.5)	0.57
Magnesium (mg/24h)	98.2 ± 78.7	87.5 ± 38.0	0.23
Citrate (mg/24h)	765 ± 578	449 ± 254	< 0.001
Oxalate (mg/24h)	20.2 ± 7.4	23.9 ± 10.4	0.02
Uric acid (mg/24h)	551 (430 - 665)	585 (438 - 726)	0.31
Phosphorus (mg/24h)	741 (498 - 923)	821 (598 - 1052)	0.58
Urea (g/24h)	18.4 (14.4 - 24.2)	20.7 (16.1 - 24.8)	0.15
Creatinine (mg/24h)	1379 (1108 - 1801)	1478 (1174 - 1892)	0.36
pH	6.2 (5.6 - 6.6)	6.1 (5.7 - 6.5)	0.15

BMI: body mass index; WC: waist circumference; Mean ± SD ; Median (IQR).

Table 2 shows the correlations between urinary and anthropometric parameters. Among SF, there was a significant positive correlation of BMI with urea, creatinine, uric acid, sodium, and oxalate excretion and of WC with urea, creatinine, uric acid, calcium, sodium, and oxalate excretion. WC was negatively correlated with urine pH in SF. Among non-SF, WC was positively correlated with urea, creatinine, uric acid, calcium, and citrate. Only 14.4% of patients had a stone analysis and among them, 81.2% were composed of either monohydrated or dihydrated calcium oxalate, 15.6% of uric acid and 3.2% of cystine.

**Table 2:** Correlation between urinary and anthropometric parameters of stone formers (SF) and non-stone formers (non-SF)

Urinary parameters	Non-SF		SF	
	BMI	WC	BMI	WC
	r	r	r	r
Urea (mg/24hs)	0.30	0.54*	0.35†	0.38†
Creatinine (mg/24hs)	0.07	0.39*	0.39†	0.39†
Uric acid (mg/24hs)	0.36	0.42*	0.49†	0.45†
Calcium (mg/24hs)	0.21	0.37*	0.14	0.18*
Citrate (mg/24hs)	0.22	0.43*	-0.01	-0.04
Sodium (mEq/24hs)	0.02	0.16	0.43†	0.39†
Oxalate (mg/24hs)	0.04	0.11	0.31*	0.31*
pH	0.10	-0.08	-0.07	-0.15*

BMI: body mass index; WC: waist circumference;

† *p* < 0.001;

* *p* < 0.05.

Regarding dietary data, to avoid underestimation, the basal metabolic rate (BMR) was calculated and matched to caloric intake obtained from the 3-day food records (data not shown in tables). The median caloric intake from food records was significantly higher than BMR for both SF (1826 Kcal [1456 - 2259] vs 1565 Kcal [1415 - 1782], *p* < 0.001) and non-SF groups (1757 Kcal [1480 - 2156] vs 1608 Kcal [1439 - 1790], *p* < 0.001). As seen in Table 3, the SF group presented a significantly higher intake of protein (assessed by PNA), fiber, and NaCl and lower intake of lipids and calcium, especially of animal origin. When comparing PNA with protein intake obtained from food records, we observed that mean PNA was significantly higher than the latter in both SF (1.8 ± 0.1 g∕kg vs 1.1 ± 0.4 g∕kg, *p* < 0.001) and non-SF (1.4 ± 0.1 g∕kg vs 1.1 ± 0.4 g∕kg, *p* = 0.04). Their DASH-style diet profile revealed lower intake of fruits, low-fat dairy items, and sweets/added sugar servings but higher intake of vegetables and nuts/seeds/legumes when compared to non-SF. Anyway, both studied groups did not disclose a dietary profile close to a DASH-style diet.

**Table 3:** Nutritional data of stone formers (SF) and non-stone formers (non-SF)

		Non-SF	SF	*p*
PNA (g/kg)		1.4 ± 0.1	1.8 ± 0.1	0.03
Lipids (% energy)		29.9 ± 9.2	25.4 ± 11.5	< 0.001
Carbohydrate (% energy)		49.6 ± 9.3	50.7 ± 8.8	0.52
Fiber (g)		17.1 ± 7.9	20.1 ± 11.2	0.02
Calcium (mg)		596 ± 292	492 ± 266	0.001
animal (mg)		437 ± 275	330 ± 227	< 0.001
vegetable (mg)		158 ± 72.0	161 ± 99.6	0.84
Phosphorus (mg)		1000 ± 33.5	961 ± 26.5	0.09
animal (mg)		632 ± 270	590 ± 350	0.18
vegetable (mg)		378 ± 171	364 ± 159	0.44
Oxalate (mg)		47.9 ± 5.5	56.2 ± 4.4	0.51
Potassium (mEq)		52.8 ± 18.7	51.3 ± 21.0	0.32
Magnesium (mg)		205 ± 71.0	195 ± 72.1	0.44
Vitamin C (mg)		61.5 ± 69.3	77.3 ± 79.6	0.48
NaCl (g)		10.1 ± 3.4	12.0 ± 5.2	0.01
*DASH-style food groups*	Recommended Servings			
Refined grains^a^	6 - 8	7.3 ± 2.4	7.8 ± 3.1	0.09
Whole grains^a^	ND	0.3 ± 0.7	0.3 ± 0.8	0.50
Vegetables^a^	4 - 5	1.4 ± 1.3	1.6 ± 1.4	0.03
Fruits^a^	4 - 5	1.4 ± 1.3	1.3 ± 1.5	0.02
Low-fat dairy^a^	2 - 3	0.6 ± 0.9	0.3 ± 0.7	< 0.001
Lean meats (red, poultry and fish) and eggs^a^	< 2	1.5 ± 0.8	1.5 ± 0.9	0.52
Nuts, seeds and legumes^b^	4 - 5	8.1 ± 7.3	12.3 ± 10.3	< 0.001
Fats and oils^a^	2 - 3	2.2 ± 1.0	2.5 ± 1.2	0.008
Sweets and added sugar^b^	< 5	24.7 ± 18.2	20.4 ± 14.6	< 0.001
Sodium (mg)^a^	2300	2810 ± 136	4110 ± 290	0.02

PNA: protein equivalent of nitrogen appearance; items reported as servings per day^a^ or per week^b^;ND - Non determined.

Table 4 shows correlation coefficients for DASH-diet food groups and lithogenic parameters in the SF group. It is important to emphasize that from 222 patients, 190 collected the 24-hr urine in the third day of the completion of the food record and the remaining 32, up to 1 week after. There was a significantly positive correlation between the group of refined grains with the urinary excretion of oxalate, sodium and uric acid, servings of vegetables with urinary potassium, fruits with urinary citrate, lean meats with urinary oxalate, sodium and uric acid, and nuts/seeds/legumes servings with urinary oxalate and potassium. We also found a significantly negative correlation between the group of low-fat dairy products with urinary oxalate, sodium, and citrate.

**Table 4:** Correlation between DASH-diet food groups and lithogenic parameters in SF group

Food groups servings	Calcium	Oxalate	Sodium	Citrate	Uric Acid	Potassium
Refined grains	0.08	0.29*	0.17*	0.05	0.16*	-0.04
Whole grains	0.07	-0.05	-0.04	0.07	0.01	-0.00
Vegetables	0.03	0.16	0.08	-0.00	-0.04	0.19*
Fruits	-0.03	0.09	-0.09	0.13*	-0.02	0.06
Low-fat dairy	0.01	-0.20*	-0.18*	-0.21*	-0.06	0.12
Lean meats and eggs	0.09	0.19*	0.13*	0.11	0.31†	0.12
Nuts, seeds and legumes	0.10	0.36*	0.26†	0.17	0.18*	0.20*
Fats and oils	-0.01	0.09	0.08	005	-0.03	-0.04
Sweets and added sugar	-0.05	-0.12	-0.03	0.03	-0.03	-0.12

† *p* < 0.001;

* *p* < 0.05.

**Figure 1:**
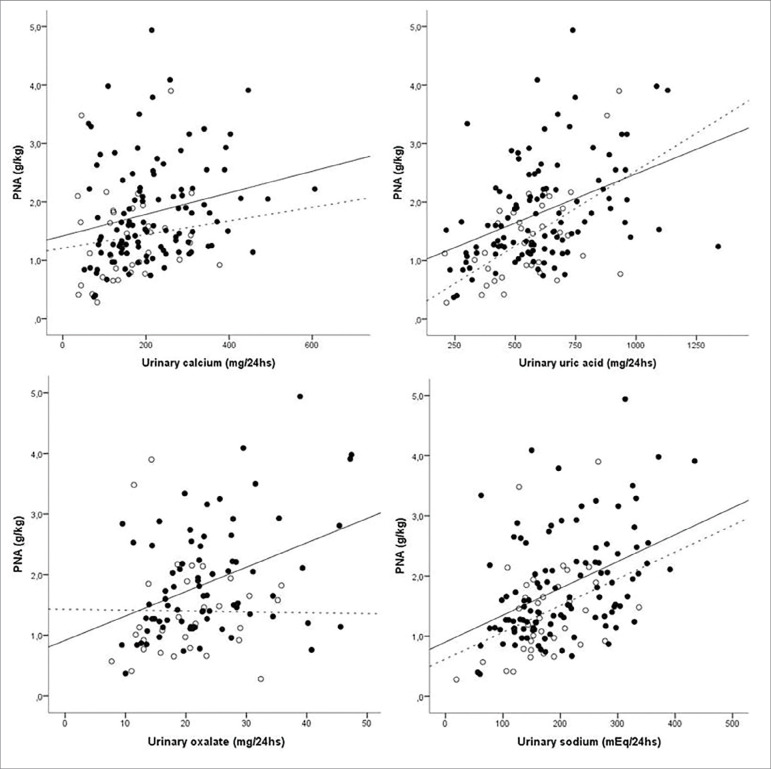
Scatterplots between PNA (protein equivalent of nitrogen appearance) and lithogenic parameters in stone formers (SF) (● ―) and non-SF (○- - -). Urinary calcium: SF r=0.28 (*p*=0.003) non-SF r=0.19 (p=0.26) / Urinary sodium: SF r=0.43(*p*<0.001) non-SF r=0.36 (*p*<0.005) / Urinary oxalate: SF r=0.35 (*p*<0.05) non-SF r=0.16 (*p*=0.37) / Uric Acid SF r=0.43 (*p*<0.001) non-SF r=0.48 (*p*=0.003).

As shown in Figure 1, PNA was significantly and positively correlated with urinary calcium, oxalate, sodium, and uric acid in SF and only with the last two urinary parameters in non-SF.

**Figure 2:**
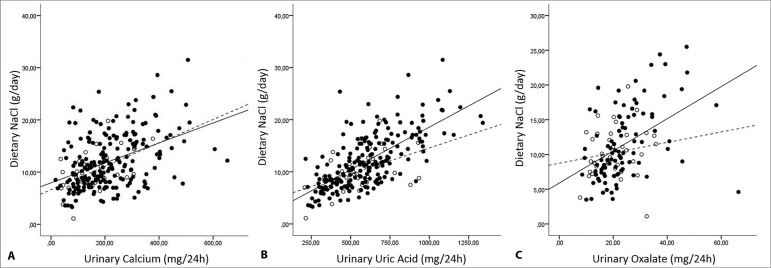
Scatterplots between dietary NaCl and lithogenic parameters in stone formers (SF) (● ―) and non-SF (○- - -). A Urinary calcium: SF r = 0.41 (*p*<0.001) non-SF r=0.54 (*p*=0.001) / B Urinary uric acid: SF r=0.69 (*p*<0.001) non-SF r=0.47 (*p*=0.003) / C Urinary oxalate: SF r=0.52 (*p*<0.001) non-SF r=0.31(*p*=0.16).

There was a significant positive correlation between NaCl intake with urinary calcium, uric acid, and oxalate among SF and with urinary calcium and uric acid in non-SF (Figure 2).

## Discussion

A DASH-style diet has been previously shown to be associated with a reduced risk of renal stone formation by increasing urinary citrate and volume^8^. The present study aimed to evaluate the dietary profile of SF and the adequacy to a DASH-style diet with their influence upon lithogenic parameters.

In the current series, SF were older, had more obese and overweight percentage, and presented higher mean serum levels of glucose and triglycerides than non-SF. Accordingly, SF had a higher prevalence of diabetes (and hypertension). Their metabolic profile showed higher urinary excretion of calcium, sodium, oxalate and lower citrate when compared to non-SF. Moreover, among SF, both BMI and WC were directly correlated with urinary oxalate and sodium. WC was positively correlated with urinary calcium and negatively correlated with urinary pH. These observations agree well with data reported by Shavit et al.^14^, which emphasized that not only obese, but also overweight stone-forming patients, presented higher urinary calcium, sodium, and oxalate. These findings highlight that both obesity and overweight affect urinary lithogenic parameters, as previously reported^3^.

Considering the recommendation of calcium intake for healthy adults to be around 1000 mg/day, the present analysis revealed a low consumption of calcium, below a mean value of 600 mg/day for both groups. These observations are in accordance with a recent systematic review about global calcium intake showing that countries in the intake categories of 400 to 500 and 500 to 600 mg/day are clustered in South America (Argentina, Bolivia, and Brazil) and scattered throughout the Far East and North Africa^15^. Irrespective of the total amount of daily calcium consumption, most of the studies show a similar or even lower calcium intake by SF when compared to healthy subjects^16^^-^ 18. Furthermore, when characterizing the dietary profile of SF from the present series, we observed that the lower mean calcium intake has been from animal sources, coupled with a trend to lower phosphorus intake from animal origin, hence suggesting a reduced consumption of dairy products. Since 1993, Curhan et al.^7^ had shown that a low calcium intake adversely affects the risk of stone formation, irrespective of its origin^19^. Current findings highlight that despite the recommendation for abolishment of calcium restriction^7^^,^ 20 a low consumption of calcium/dairy products by SF remains hitherto a counterproductive dietary habit.

In addition, in the present study, we observed a higher oxalate excretion by SF, albeit within normal range. Given that oxalate intake did not differ between groups, it seems that the lower calcium intake might have contributed to increase urinary oxalate, as less free calcium is available to bind intestinal oxalate, increasing its absorption^7^^,^ 21. The contribution of dietary oxalate to oxaluria is known to be highly dependent on calcium intake, since higher oxalate intake does not increase the risk of stone formation when calcium intake is adequate^22^, reinforcing the need to balance the intake of both. Accordingly, we did find an inverse association between low-fat dairy products intake with urinary oxalate (Table 4). In the present sample of SF, and in previous studies from our group^18^, oxalate intake had been low and within the recommendations of The American Dietetic Association (< 60 mg/day) but far below from epidemiological data from USA, around 200 mg/day^23^.

Paradoxically, one of the intriguing findings of the present study was that the mean 24-h urinary volume was higher among SF compared to non-SF, probably reflecting previous advice to increase their fluid intake from other doctors and health care professionals or even from the media.

A higher protein intake by SF was found when compared to non-SF, in accordance with other investigators^17^. On the other hand, although protein is expected to alter many urinary parameters related to kidney stone formation, several studies did not observe a higher protein consumption among stone-forming patients^16^^,^ 24. Such discrepancies may be attributed to the current use of PNA, which has not been employed in any of the above-mentioned studies. The significant difference between PNA and protein intake calculated from food records in the present series could be due to PNA reflecting the protein intake from one day as opposed to the mean of 3 days from the records. Nevertheless, similar differences were already described by other authors^25^. Anyway, urine nitrogen is a much better surrogate marker of dietary protein than estimates from dietary records^26^. We observed a positive correlation between PNA with urinary calcium, which can be ascribed to the acid load of protein^27^, or not^28^. The current increased PNA by SF was not accounted for by the consumption of meat.

The food diary revealed a very low or no consumption at all of seeds and nuts. Therefore, the largest contribution to a significantly higher consumption of this food group by SF might have been from legumes, especially beans. A high daily consumption of beans is indeed a very frequent part of the daily eating habits of the Brazilian population, disclosed by 70% of individuals^29^. In addition to guaranteeing a good portion of legumes, the consumption of beans further contributes to the increase of dietary potassium, fiber, and vegetable protein. On the other hand, it may provide a high amount of oxalate as well^13^^,^ 30. Although Ferraro et al.^31^ did not find an increased risk for stone formation by vegetable protein, it is important to emphasize that the amount of protein intake of SF in the present series was more than twice above the IOM recommendation for total protein intake of 0.8 g/kg/day. Anyway, the possibility that processed and non-lean meats had contributed to the increased PNA cannot be ruled out since the 24-hr urine collection was not obtained on the same day of the dietary record in all patients.

The evaluation of the DASH-diet food groups revealed that the consumption of lean meats/eggs did not differ between SF and non-SF. However, the lean meats/eggs food group and PNA were both positively correlated with oxalate excretion only among SF. Noteworthy, the legumes food group was also directly correlated with oxaluria. These observations are in accordance with data by Nguyen et al.^32^ that showed that one third of stone-forming patients are sensitive to meat protein intake in terms of oxalate excretion. Although theoretically the oxaluric effect of protein is linked to the intake of hydroxyproline, an amino acid found in meat, epidemiologic data showed no relationship between animal protein intake and oxalate excretion^33^. The present findings suggest that a high consumption of non-lean/processed meat, beans, or both were responsible for increasing oxalate excretion. Another observed positive correlation was between the DASH-diet food groups of refined grains, lean meats/eggs, and legumes with urinary uric acid among SF. Finally, urinary uric acid correlated with PNA in both groups, as reported elsewhere^31^.

In the present series, SF presented a higher NaCl intake than non-SF, which correlated directly with urinary calcium, uric acid, and oxalate. An elevated salt consumption by SF has been previously disclosed by our group^34^^,^ 35 and others^17^. The effect of Na intake on increasing calcium excretion is well established^36^ and a cross-sectional study found that SF in the highest quartiles of urinary sodium excreted 37 mg/day more urinary calcium than participants in the lowest quartile^37^. Moreover, we have previously shown that a high NaCl intake is related to bone loss among stone formers^34^. The reduction of oxalate excretion by decreasing sodium intake has been previously ascribed to a reduction in intestinal oxalate absorption^38^. More recently, it has been shown that urinary uric acid was significantly decreased by a low-salt diet^39^.

In the present study, none of the groups presented a DASH-style diet pattern. SF only achieved the recommended intake suggested by DASH diet with respect to four food groups: refined grains, lean meats/eggs, legumes, and fats/oils. The average intake of vegetables, fruits, whole grains, and low-fat dairy products did not even reach the minimum recommendation of 2 portions per day for the first two food groups and 1 portion per day for the last two. It is also important to emphasize that the average intake of Na by SF was almost twice the amount advised by DASH diet and the habitual consumption of sweets and added sugar were four-fold higher than the recommendation of 5 portions/week. Curiously, the consumption of the latter among SF was significantly lower when compared to non-SF, an observation that can be explained by the higher prevalence of diabetes among SF.

Despite of an inadequate consumption of fruits, the current data showed a positive correlation between fruit consumption and citrate excretion among SF, as expected^10^^,^ 40. In addition, albeit the intake of vegetables was also below the recommendation for both groups, the significantly higher intake detected among SF was still enough to be positively associated with urinary potassium. On the other hand, dietary potassium detected by the dietary record did not differ between groups, which might have been the result of a higher intake of vegetables but a lower amount of fruits by SF.

Some limitations of our study should be pointed out. Nutrients and DASH diet food groups were derived from a self-reported 3-day food diary, which is known to provide underreported data and errors in estimation of portion sizes. Oxalate might have been underreported since the present analysis was performed including only the available foods provided on the Harvard website^13^. Groups were not age-matched, but the present results were statistically adjusted through GLM. One of the strengths of this research was the evaluation of a dietary pattern among stone-forming patients using a 3-day food record since most of the previous articles had focused on macro and micronutrients intake evaluated by food frequency questionnaires, which is not quantitatively precise. It is also important to highlight the relevance of this study for disclosing a possible DASH-style diet in a different western nation.

In summary, we detected a higher prevalence of obesity in the current series of SF, and a positive correlation between WC and urinary calcium, sodium, uric acid, and oxalate and negative with urinary pH. Although SF achieved the recommended intake suggested by DASH diet regarding refined grains, lean meats/eggs, legumes, and fats/oils, they still consume less calcium, and more salt and protein than the recommendation, not reflecting an ideal DASH-style diet pattern.
